# Analytical and statistical consideration on the use of the ISAG-ICAR-SNP bovine panel for parentage control, using the Illumina BeadChip technology: example on the German Holstein population

**DOI:** 10.1186/s12711-014-0085-1

**Published:** 2015-02-05

**Authors:** Ekkehard Schütz, Bertram Brenig

**Affiliations:** Institute of Veterinary Medicine, Burckhardtweg 2, D-37077 Göttingen, Germany

## Abstract

**Background:**

Parentage control is moving from short tandem repeats- to single nucleotide polymorphism (SNP) systems. For SNP-based parentage control in cattle, the ISAG-ICAR Committee proposes a set of 100/200 SNPs but quality criteria are lacking. Regarding German Holstein-Friesian cattle with only a limited number of evaluated individuals, the exclusion probability is not well-defined. We propose a statistical procedure for excluding single SNPs from parentage control, based on case-by-case evaluation of the GenCall score, to minimize parentage exclusion, based on miscalled genotypes. Exclusion power of the ISAG-ICAR SNPs used for the German Holstein-Friesian population was adjusted based on the results of more than 25 000 individuals.

**Results:**

Experimental data were derived from routine genomic selection analyses of the German Holstein-Friesian population using the Illumina BovineSNP50 v2 BeadChip (20 000 individuals) or the EuroG10K variant (7000 individuals). Averages and standard deviations of GenCall scores for the 200 SNPs of the ISAG-ICAR recommended panel were calculated and used to calculate the downward *Z*-value. Based on minor allelic frequencies in the Holstein-Friesian population, one minus exclusion probability was equal to 1.4×10^−10^ and 7.2×10^−26^, with one and two parents, respectively. Two monomorphic SNPs from the 100-SNP ISAG-ICAR core-panel did not contribute. Simulation of 10 000 parentage control combinations, using the GenCall score data from both BeadChips, showed that with a *Z*-value greater than 3.66 only about 2.5% parentages were excluded, based on the ISAG-ICAR recommendations (core-panel: ≥ 90 SNPs for one, ≥ 85 SNPs for two parents). When applied to real data from 1750 single parentage assessments, the optimal threshold was determined to be *Z* = 5.0, with only 34 censored cases and reduction to four (0.2%) doubtful parentages. About 70 parentage exclusions due to weak genotype calls were avoided, whereas true exclusions (n = 34) were unaffected.

**Conclusions:**

Using SNPs for parentage evaluation provides a high exclusion power also for parent identification. SNPs with a low GenCall score show a high tendency towards intra-molecular secondary structures and substantially contribute to false exclusion of parentages. We propose a method that controls this error without excluding too many parent combinations from the evaluation.

**Electronic supplementary material:**

The online version of this article (doi:10.1186/s12711-014-0085-1) contains supplementary material, which is available to authorized users.

## Background

Currently, parentage control in cattle is mainly based on short tandem repeats (STR), but is moving towards single nucleotide polymorphism (SNP)-based methods [1]. In order to harmonize the latter, the International Society for Animal Genetics and the International Committee for Animal Recording (ISAG-ICAR) have defined a panel of 200 SNPs for parentage control [2,3]. This panel consists of 100 core and 100 additional SNPs, mainly selected for high minor allelic frequencies (MAF) to facilitate a high exclusion probability in a variety of cattle breeds. Recommendations on the thresholds to accept and exclude parentage are proposed [4].

For most STR-based methods, quality control (QC) relies on statistical procedures, such as running samples with a known accuracy as controls and assuming that unknown samples will perform the same way. In contrast, for SNP-based genotyping methods, QC measures are usually specific to each array-based technology. For example, the Illumina bead chip system applies a GenCall score, which is a measure of goodness of the call for any individual SNP of a sample on a chip [5]. These measures are mainly used to censor the data for which the probability that the call is correct is low, based on an overall threshold. In most cases, when using high-density SNP data, a limited number of miscalled genotypes can be tolerated. For example, in breeding index calculations, the effect of SNP genotype miscalls that occur with a low frequency, will be small and thus will not influence the overall result of the breeding value of an individual cattle. Therefore, based on cost/benefit considerations, a low threshold is used to gain as much information as possible on a more population-based point of view. Nevertheless, for parentage control, including samples with low genotype calls i.e. a higher error probability has several disadvantages, since they can lead to false exclusions or doubtful results [6]. It has been suggested to discard results with a GenCall score lower than 0.7, if highly reliable results are necessary for decision making [7,8]. Other technical and population genetics considerations, about the usefulness of SNPs for parentage control have been discussed [9]. In parentage control, two to three samples are examined, for which the sum of the calling errors will - based on theory - lead to a higher total error with an increased likelihood of false exclusions. To control this error, we propose to use the technical error value (GenCall score) for each SNP of the 200-SNP panel on an individual (per sample) basis, when used for parentage control. This facilitates a process-based rather than a statistical quality control. To prove this concept, large-scale simulations and applications to real data were performed.

## Methods

The Illumina BovineSNP50 v2 and EuroG10k BeadChips were used to genotype a German Holstein-Friesian bovine population according to the manufacturers’ recommendations. For both BeadChips, the cluster files supplied by Illumina were used for GenCall and re-clustering was done once for such SNPs with an overall call rate lower than 97%. The new cluster file was stored and used without further modification throughout the study. Overall, based on the manufacturer’s recommendations, genotypes with a GenCall score greater than 0.15 were considered called. Genotype data were obtained for 20 000 and 7000 Holstein-Friesian individuals using the BovineSNP50 v2 and the EuroG10k BeadChip, respectively. From the genotyping data available on the respective BeadChip version, the data of the 200-SNP ISAG-ICAR panel, which consists of 100 core SNPs and 100 additional SNPs (the latter to be used for cases, for which paternity control using the 100 core SNPs alone yields a doubtful result) were extracted and the average GenCall score ($\overline{X}$) and the standard deviation (SD) were calculated for each SNP and each chip type, separately [See Additional file 1 Table S1]. In order to combine data from both BeadChips, each GenCall score was Z-transformed as follows:

$$ {Z}_{s,i}=\frac{G{C}_{s,i,n}-\overline{X_{i,n}}}{S{D}_{i,n}}, $$

where *GC* is the GenCall score, *n* is the chip type, *i* is the SNP locus and *s* is the sample.

The total error in genotype calling for each SNP in any parentage evaluation was estimated using a simple error summation formula:

$$ {Z}_{s, total}=\sqrt{\sum_{i=1}^n{Z}_{s,i}^2}, $$

where *Z*_*s*_*,*_*i*_ is from the offspring and one (*n* = 2) or two (*n* = 3) parents tested, and the *s* denotes the SNP tested.

Since error contribution is considered as a one-sided phenomenon (calls with a better than average GenCall score do not improve the total error), *Z*-values greater than 0 were set to 0 for total error calculation.

Ten thousand random combinations from our real data were used to simulate the censoring effect of using the *Z*_*s,total*_ values for excluding SNPs from data evaluation. For this, *Z*_*s,total*_ for each SNP was calculated from *Z*_*s*_*,*_*i*_ for each random combination and the number of SNPs above the threshold value for *Z*_*s,total*_ was recorded by sliding the threshold from 2.67 to 6 in $0.3\overline{3}$ intervals.

The threshold for censoring a SNP from parentage evaluation was set to 3.66, which is equivalent to an error control rate of 2.5% when corrected for multiple testing using the Bonferroni procedure.

Minor allelic frequencies (MAF) of SNPs in the 200-SNP panel in the German Holstein-Friesian population were calculated for SNPs with a GenCall threshold greater than 0.15, with the exception of SNP ARS-USMARC-Parent-DQ916057-rs29009979, which is not present on the BovineSNP50 v2 BeadChip. Therefore, in this case, the MAF was calculated based on the data obtained from the 7000 Holstein-Friesian individuals genotyped using the EuroG10k BeadChip.

The guanine-cytosine (GC) content of the 60-bp genomic sequence on either side of each SNP was calculated and compared to the average GenCall score using a linear correlation model.

Standard formulas to convert the allelic frequencies into probability *P* of parentage exclusion were used [10].

For one parent:

$$ P=1-4{\displaystyle \sum_{i=1}^n}{p}_i^2+2{\left({\displaystyle \sum_{i=1}^n}{p}_i^2\right)}^2 $$

$$ +4{\displaystyle \sum_{i=1}^n}{p}_i^3-3{\displaystyle \sum_{i=1}^n}{p}_i^4 $$

For two parents:

$$ P=1+4{\displaystyle \sum_{i=1}^n}{p}_i^4-4{\displaystyle \sum_{i=1}^n}{p}_i^5-3{\displaystyle \sum_{i=1}^n}{p}_i^6-8{\left({\displaystyle \sum_{i=1}^n}{p}_i^2\right)}^2 $$

$$ +8\left({\displaystyle \sum_{i=1}^n}{p}_i^2\right)\left({\displaystyle \sum_{i=1}^n}{p}_i^3\right)+2{\left({\displaystyle \sum_{i=1}^n}{p}_i^3\right)}^2 $$

The probabilities were calculated for each locus tested, where *p*_*i*_ is the frequency of allele *i*, *n* the number of alleles (usually two alleles per SNP). The total exclusion power is calculated by combining all *P* values of the tested loci as follows:

$$ P=1-\left(1,-,{P}_1\right)\left(1,-,{P}_2\right)\left(1,-,{P}_3\right)\dots \left(1,-,{P}_k\right), $$

where k is the number of loci used.

All calculations were performed using either standard UNIX commands or Microsoft Excel^™^.

## Results

**Figure 1:**
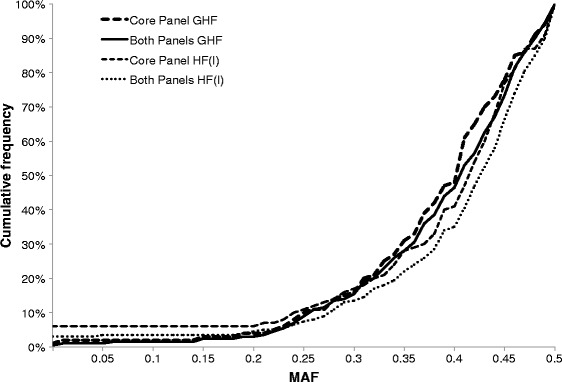
**Comparison of minor allelic frequencies given in the initial report (Illumina) with those estimated in the German Holstein population with the 100-SNP core panel (Core) and 200-SNP panel (Both).** HF(I): calculated using MAF data from the manufacturer’s pre-evaluation in Holstein-Friesian; GHF: calculated using the estimated MAF data in the German Holstein-Friesian population.

**Figure 2:**
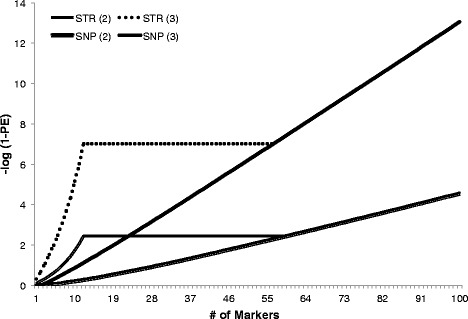
**Comparison of exclusion probabilities of the 12 ISAG short tandem repeats with the 100 core SNPs.** Lines for short tandem repeats (STR) are extended to the cross-line with SNPs, showing that with about 55 SNPs similar PE values are obtained than with STR. For this chart, both STR and SNPs were ranked for PE (high to low); (2) = Two individuals (Offspring and one parent) analyzed; (3) = Offspring and both parents analyzed.

MAF in the German Holstein-Friesian population differed slightly from the MAF provided by Illumina for Holstein Friesian cattle as shown in Figure 1. The three following SNPs of the 200-SNP panel are not useful for the German Holstein-Friesian population i.e. ARS-USMARC-Parent-DQ786764-no-rs and ARS-USMARC-Parent-EF034087-no-rs are monomorphic and ARS-BFGL-NGS-72471 has a MAF of only 5.8%. For both recommended panels (100 core SNPs and 100 additional SNPs) of the ISAG-ICAR SNP panel, 85% of the SNPs have a MAF greater than 0.3, which results in an overall high exclusion probability. The overall probability of non-exclusion (1-PE) of the 100 core SNPs is 1.4×10^−5^ and 1.4×10^−10^ using the 200 SNPs of the additional panel if one parent is interrogated. For a complete trio, the values are 2×10^−13^ and 7.2×10^−26^, respectively. Figure 2 shows the comparison of PE values that are achieved with SNPs in comparison to the ISAG-defined STR markers. These values also underline that even if one parent is unknown, the 200-SNP panel will unequivocally result in the identification of a single individual cattle.

Average GenCall scores from both the BovineSNP50v2 and the EuroG10k BeadChips were calculated and compared to a censoring of SNPs, based on the recommended value of 0.7 [8]. For the core panel, 10 SNPs (EuroG10k) and 20 SNPs (BovineSNP50v2 BeadChip) would be lost (95% confidence value) while for the 200-SNP panel, 20 SNPs (EuroG10k) and 29 SNPs (BovineSNP50v2 BeadChip) would be lost. A highly significant inverse correlation was found between the upper (97.5%) confidence limits of GenCall scores (97.5% confidence limit) and the GC-content of the surrounding genomic sequence (BovineSNP50v2 BeadChip: GenCall score = −0.38 × GC-content + 1.07, F-test - p=6.5×10^−9^; EuroG10k bead chip: GenCall score = −0.24 × GC-content + 1.02, F-test - p=5.5×10^−6^). Using a GenCall score of 0.9 and a GC-content of 46% as delimiters, the Fisher’s exact p value for e.g. the BovineSNP50v2 BeadChip was 5.9×10^−9^. Sequences with a high GC-content are prone to form secondary intra-molecular structures (secondary structures) [11]. Such secondary structures were determined for the SNP with the lowest average GenCall score in both BeadChips, i.e. ARS-USMARC-Parent-DQ837645-rs29015870, and a stable structure (deltaG: −12.3 kcal/mol) was demonstrated [See Additional file 2 Figure S1].

In order to evaluate the losses of resolvable parentages due to censoring of SNPs by integrating a concept of individual QC into the parentage control, a simulation with different threshold values, based on the actual dataset was performed. Ten thousand random combinations for one and two parents were calculated and the GenCall score was used to calculate the total error as indicated above. The number of combinations for both one and two parents that had to be excluded because the number of interrogated SNPs was less than recommended (90 for one and 85 for two parents) was ~2.5%, when the threshold for total error was set to *Z*_*total*_ = 3.66, which is close to the value obtained from the Gaussian probability function [See Additional file 3 Figure S2].

The *Z*_*total*_ was finally tested on 1750 known real parentage control combinations in the same dataset. The dataset contained only combinations with one interrogated parent which is the usual case in routine laboratory diagnosis compared to cases where both parents are questionable. Data without considering the GenCall score were used as side-by-side comparison. Overall, 62 (3.5%) combinations did not reach the necessary number of interrogated SNPs based on the recommended minimum GenCall score of 0.15 (n = 90 SNPs), but it should be noted that the missing SNP on the BovineSNP50v2 BeadChip already contributes in many cases. The influence on the parentage evaluation is substantial, since the number of doubtful cases decreased from more than 400 to only less than 10; the number of exclusions was also greatly reduced to 35 (110 without censoring weak calls). Random censoring of an equivalent number of SNPs did not alter the results compared to unfiltered data.

**Figure 3:**
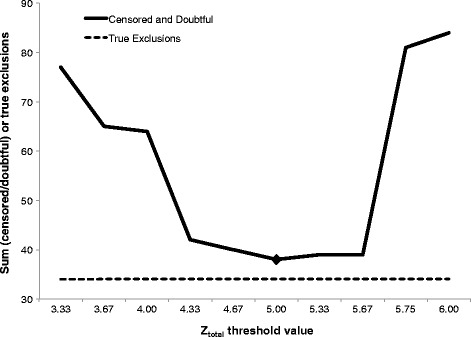
**Optimization of total** ***Z*** **-value using a risk/reward approach.**

**Figure 4:**
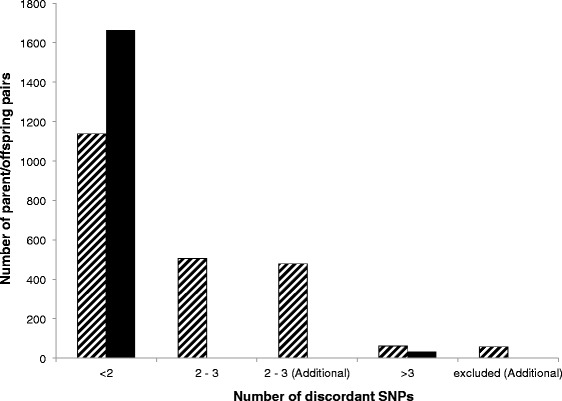
**Effect of error control at a combined** ***Z*** **-value of 5.0 on frequencies of parent-offspring pairs according to numbers of conflicting genotypes.** Hatched bars correspond to un-censored numbers and solid bars correspond to results obtained by applying the error control algorithm.

In a final risk/reward analysis the best *Z*_*total*_ value was chosen by minimizing the number of unsolved and doubtful parentages in parallel. Figure 3 shows that the optimal *Z*_*total*_ threshold value was equal to 5.0. Using this *Z*_*total*_ value, only 34 combinations were censored, whereas the number of remaining doubtful parentages was minimized (four cases). The number of true (verified) parentage exclusions, which were part of the evaluation was constant with 34 cases over the whole range of threshold values, which indicates that the approach does not result in erroneous parentage acceptances. The effect of this approach is shown in Figure 4, where the unfiltered parentage results are displayed in comparison to the results after applying the error control algorithm, based on a *Z*_*total*_ of 5.0 as delimiter. Interestingly, a cluster of five SNPs could be defined that had a high rate of being excluded in both BeadChips. This suggests that re-designing the respective probes to optimize their detection in genotyping analyses might be advisable. Those SNPs that performed poorly and therefore, were most often censored from the analyses are listed in Table 1.

**Table 1:** **Low performing SNPs within the core and additional ISAG-ICAR panel**

**SNP-name**	**Panel**	**Average GC-score (50k)**	**Average GC-score (10k)**	**Censored SNP (%)**
***ARS-USMARC-Parent-DQ786762-rs29010772***	C	0.62	0.66	26.4
ARS-USMARC-Parent-EF093511-rs29012316	C	0.66	0.79	7.2
ARS-BFGL-NGS-11383	A	0.79	0.78	6.2
***Hapmap52240-rs29013844***	A	0.64	0.96	3.6
***ARS-USMARC-Parent-DQ837645-rs29015870***	C	0.75	0.55	3.2
***Hapmap46653-BTA-47447***	A	0.87	0.64	2.9
ARS-USMARC-Parent-DQ404150-rs29012530	C	0.84	0.82	2.3
***ARS-USMARC-Parent-EF034083-rs29018286***	C	0.74	0.79	2.0
ARS-BFGL-NGS-42505	A	0.85	0.92	1.9
ARS-USMARC-Parent-DQ404151-rs29019282	C	0.59	0.69	1.8
ARS-USMARC-Parent-DQ846693-rs29017621	C	0.85	0.83	1.3
ARS-BFGL-NGS-118319	A	0.64	0.74	1.2
Hapmap46550-BTA-103548	A	0.96	0.85	1.1
ARS-USMARC-Parent-DQ839235-rs29012691	C	0.86	0.86	1.0
UA-IFASA-5034	A	0.83	0.75	1.0
ARS-USMARC-Parent-DQ837644-rs29010468	C	0.83	0.89	0.4

SNPs that were most often censored in parentage analysis on both chip types ranked in order of censored parentages (highest first); SNPs that performed poorly on both chip types are marked by bold/italic characters; GC score = GenCall score; 50k = BovineSNP50v2 BeadChip; 10k = EuroG10kv2 BeadChip; C = core panel; A: additional panel.

## Discussion

The usefulness of SNPs for parentage control is substantiated by the high exclusion power of the ISAG-ICAR panel SNPs [12,13]. Nevertheless, it seems impossible to manually check each of the 200 data points for any given sample going into parentage control. Thus, it is desirable to establish a more automated process control to avoid false exclusion due to genotype miscalls [14]. Such miscalls are inherent to the method applied and cannot be avoided. We showed that the goodness of calls of SNPs is strongly influenced by the secondary intra-molecular structures of the genomic sequence surrounding each SNP.

We have theoretically and practically evaluated a concept of error minimization by integrating the individual GenCall score that is given for each SNP call for any given sample into the SNP-based parentage control workflow. We show that this leads to an almost complete elimination of false exclusions and doubtful results. Especially the latter is meaningful, since according to the ISAG-ICAR guidelines, such doubtful results would require callbacks and case-by-case discussions. It is conceivable that a re-clustering of each run would also lead to better calls (to a certain extent), but such a strategy does not seem to be feasible under accreditation guidelines, since it would require an enormous documentation effort.

The method of error control proposed here is suitable to minimize erroneous parentage exclusions and doubts by pre-censoring only weak genotype calls, an approach that can be automated in the software, or can be done by inspection of the *Z*-values performed by the investigator.

A discussion is probably needed on how the measurable underlying analytical error can be mirrored in planned databases for international parentage evaluations. This seems essential to avoid later issues on both parentage control and search that include low-quality SNP data that are likely to be erroneous.

Overall, the ISAG recommendations can be debated, however, currently, they are based on (1) number of SNPs, that are used (a minimum of 90 for one parent and 85 for two parents) and (2) number of conflicting genotypes. These considerations are mostly based on simulations without special consideration of the contribution of a locus for a breed and the achievable reliability. There is no reason to use monomorphic SNPs, since it does not matter whether they are genotyped or not. Finally, given the very high PE of SNP-based parentage control (Figure 2), it may be better to use the achieved maximum PE for an individual parentage control as criterion, instead of a fixed number of SNPs (85 or 90) as requirement limit. Based on the same argument of high PE, it seems reasonable to eliminate SNPs that cannot be reliably genotyped from the panel, since those would not contribute to improve PE.

## Conclusions

Although, currently, parentage control in cattle is mainly done by STR-typing, in the near future it will be replaced by SNP-based methods due to the availability of SNP genotypes from genomic selection. However, the acceptance of accurately determined SNP genotypes in genomic selection (GenCall scores) differs from the requirements for parentage control. Therefore, a re-evaluation of the statistical and quality criteria is necessary.

In the German Holstein-Friesian population, the recommended core and additional ISAG-ICAR panel contains two SNPs that are practically monomorphic and one SNP with a MAF of 5.8%, which renders them uninformative for parentage control in this population. Although the overall PE is high for both panels, inherent low GenCall scores for several SNPs, e.g. due to the formation of secondary intra-molecular structures in GC-rich sequences, will lead to a loss of data based on the recommended threshold of 0.7 for high accuracy data. In contrast, maintaining a GenCall score of 0.15 will result in an increased number of rejected or doubtful parentages due to miscalled genotypes. Censoring only those SNPs with a combined (offspring and parents) *Z*-transformed downward GenCall score of > 5.0 as threshold calculated for both BeadChips used for parentage control, the number of rejected and doubtful parentages can be substantially reduced without affecting the true exclusions.

## Additional files

Additional file 1: Table S1. Average GenCall scores and standard diviations of 100/200 SNPs of the ISAG-ICAR parentage control panels. Description: GC-score = GenCall score; 50k = BovineSNP50v2 BeadChip; 10k = EuroG10kv2 BeadChip. Additional file 2: Figure S1. Comparison of an example of a SNP with an average GenCall score (upper panel) to the SNP-locus with the lowest GenCall score (lower panel). Description: The cluster separation and the predicted secondary structure of the region surrounding the SNP-locus are shown [14]. A) Example of a SNP cluster plot with good separation. B) Cluster plot of ARS-USMARC-Parent-DQ837645-rs29015870. Additional file 3: Figure S2. Results from 10 000 simulations of parentage analyses with respect to censoring according to the used *Z*-values that were used as threshold. Description: LD = EuroG10v2 bead chips; 50k = BovineSNP50Kv2 BeadChip.
