# Supplementary figures and images for: Analytical and statistical consideration on the use of the ISAG-ICAR-SNP bovine panel for parentage control, using the Illumina BeadChip technology: example on the German Holstein population
Source: Genet Sel Evol. 2015 Feb 5;47(1):3. doi: 10.1186/s12711-014-0085-1 (PMC4318447; doi:10.1186/s12711-014-0085-1)

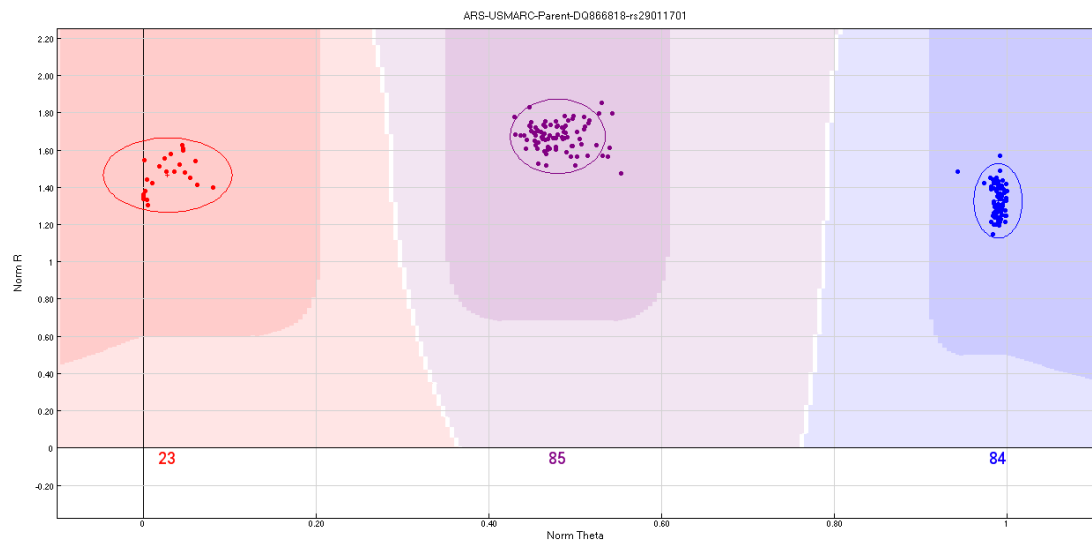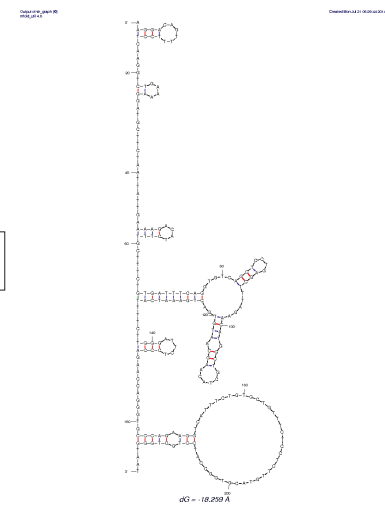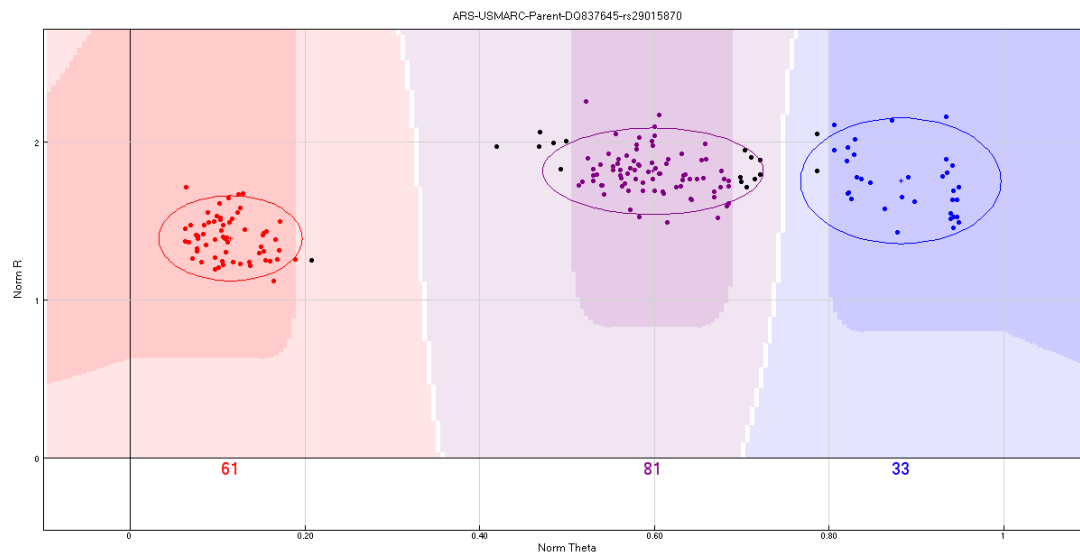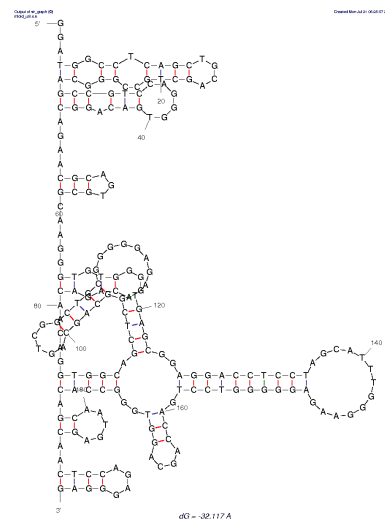

Supplement: Additional file 2: Figure S1. — Comparison of an example of a SNP with an average GenCall score (upper panel) to the SNP-locus with the lowest GenCall score (lower panel). Description: The cluster separation and the predicted secondary structure of the region surrounding the SNP-locus are shown [14]. A) Example of a SNP cluster plot with good separation. B) Cluster plot of ARS-USMARC-Parent-DQ837645-rs29015870. [file 12711_2014_85_MOESM2_ESM.pdf]

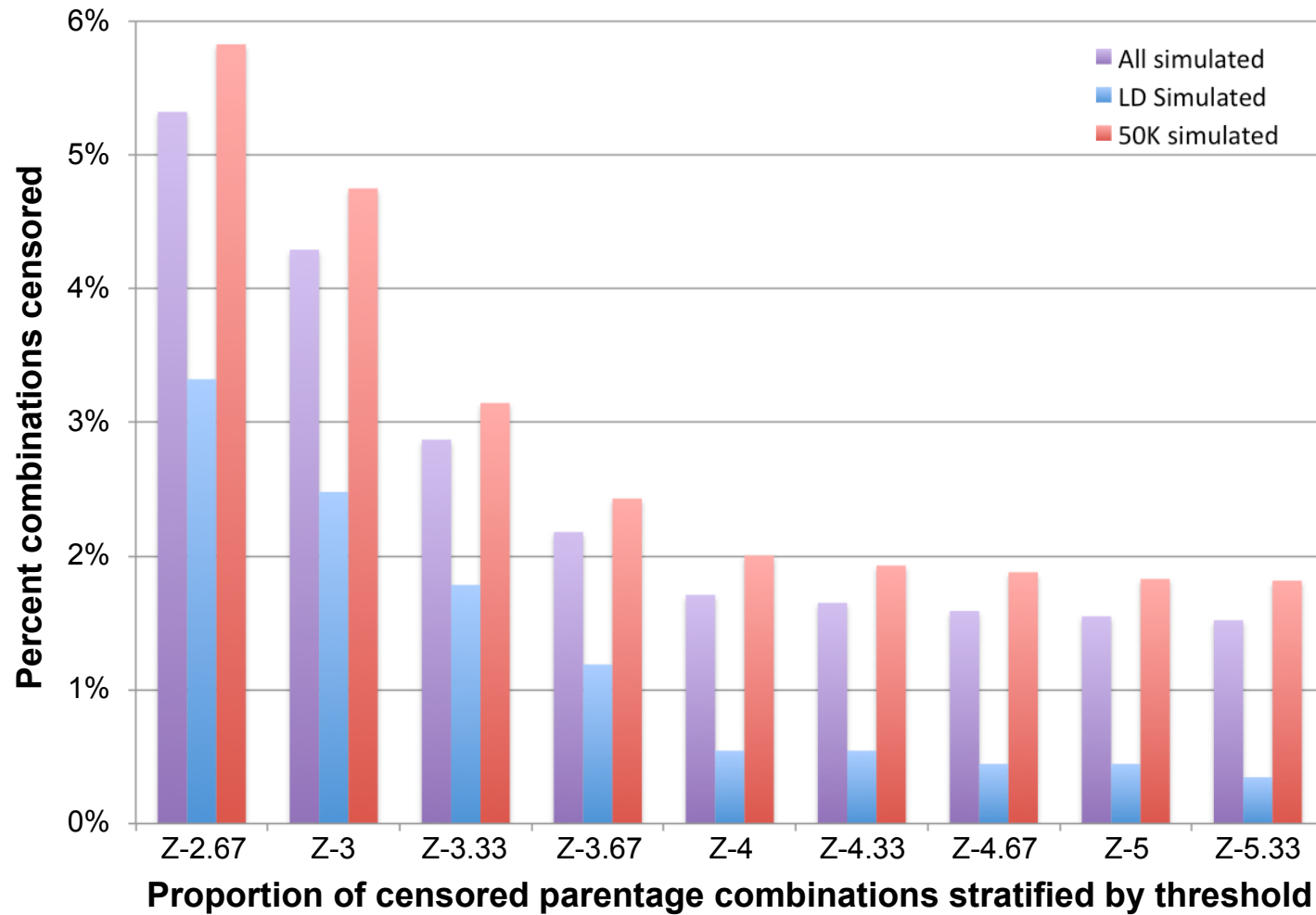

Supplement: Additional file 3: Figure S2. — Results from 10 000 simulations of parentage analyses with respect to censoring according to the used Z-values that were used as threshold. Description: LD = EuroG10v2 bead chips; 50k = BovineSNP50Kv2 BeadChip. [file 12711_2014_85_MOESM3_ESM.pdf]
